# Particles Containing Cells as a Strategy to Promote Remyelination in Patients With Multiple Sclerosis

**DOI:** 10.3389/fneur.2020.00638

**Published:** 2020-07-07

**Authors:** Jorge Matías-Guiu, Jordi A. Matías-Guiu, Paloma Montero-Escribano, Juan A. Barcia, Alejandro A. Canales-Aguirre, Juan C. Mateos-Diaz, Ulises Gómez-Pinedo

**Affiliations:** ^1^Department of Neurology, Institute of Neurosciences, IdISSC, Hospital Clínico San Carlos, Universidad Complutense de Madrid, Madrid, Spain; ^2^Laboratory of Neurobiology, Institute of Neurosciences, IdISSC, Hospital Clínico San Carlos, Universidad Complutense de Madrid, Madrid, Spain; ^3^Department of Neurosurgery, Institute of Neurosciences, IdISSC, Hospital Clínico San Carlos, Universidad Complutense de Madrid, Madrid, Spain; ^4^Unidad de Evaluación Preclínica, Unidad de Biotecnología Médica y Farmacéutica, Centro de Investigación y Asistencia en Tecnología y Diseño del Estado de Jalisco, Guadalajara, Mexico; ^5^Biotecnología Industrial, Centro de Investigación y Asistencia en Tecnología y Diseño del Estado de 12 Jalisco, CIATEJ, Zapopan, Mexico

**Keywords:** multiple sclerosis, biomaterials, oligodendrocyte progenitor cells, oligodendrocytes, demyelination, remyelination

## Abstract

The repair of demyelinated lesions is a key objective in multiple sclerosis research. Remyelination fundamentally depends on oligodendrocyte progenitor cells (OPC) reaching the lesion; this is influenced by numerous factors including age, disease progression time, inflammatory activity, and the pool of OPCs available, whether they be NG2 cells or cells derived from neural stem cells. Administering OPCs has been proposed as a potential cell therapy; however, these cells can only be administered directly. This article discusses the potential administration of OPCs encapsulated within hydrogel particles composed of biocompatible biomaterials, via the nose-to-brain pathway. We also discuss conditions for the indication of this therapy, and such related issues as the influence on endogenous remyelination, migration of OPCs to demyelinated areas, and the immune response, given the autoimmune nature of multiple sclerosis. Chitosan and derivatives constitute the most promising biomaterial for this purpose, although these issues must be addressed. In conclusion, this line of research may yield an alternative to the remyelinating drugs currently being studied.

## Introduction

Multiple sclerosis (MS) is a chronic, inflammatory, autoimmune disease in which an environment inhibiting the development of myelin-producing cells hinders repair of the myelin sheaths around demyelinated axons. Demyelination alters the conduction of neural impulses; impairment of the capacity for remyelination results in axonal degeneration, eventually leading to neuronal degeneration (1). Despite the development in recent years of numerous drugs targeting the immune mechanisms that cause inflammation, which has reduced the risk of sequelae, no drug has been found that promotes myelin repair; therefore, a central objective in current research is to design novel therapeutic strategies for remyelination (2, 3). Despite widely held opinion, the central nervous system (CNS) does have the capacity for remyelination, which has been observed in histopathological analysis of MS plaques and through neuroimaging (4–6); however, the sheaths produced can be thinner than normal, and sequelae and symptoms arising from conduction alterations are not prevented (7). This remyelination capacity is reduced or lost with age; while it is observed in early stages of relapsing-remitting MS, it is much diminished in the progressive stage of the disease. It therefore shows an inverse relationship with MS progression, and has been associated with immune activity (8) and with alterations to innate and adaptive immunity, which are reported to reduce the effectiveness of remyelination in animal models of focal demyelination; myelin repair depends on the state of activation of macrophages and microglia (9). Remyelination capacity is lost without a reduction in the number of oligodendrocyte progenitor cells (OPC), although there is a reduction in the efficiency of OPC differentiation into myelin-producing oligodendrocytes (OL) (10). In theory, effective remyelination requires: [1] the presence of OPCs in demyelinated plaques, through migration to the area of the active lesion; [2] an environment favoring OPC differentiation into OLs; [3] axons in suitable conditions for remyelination (i.e., not undergoing or having undergone a process of neurodegeneration); and [4] action of a series of signaling pathways enabling differentiation by altering OPCs, OLs, and axons in order to enable myelin production by OLs (11–14). However, several studies suggest that remyelination is not always necessary to the survival of demyelinated axons (15). The combination of both benefits may therefore be necessary to maximize the therapeutic potential of OPCs for application in clinical practice.

Work of the lasts decades past century, have explored implant tissue and oligodendrocytes, pioneering cell therapy, as a resource to promote remyelination and the search for the best cell type for this purpose (16, 17). Several types of cells can act as OPCs, but we should fundamentally consider 2 cell subtypes: NG2 cells, originating in embryonic development and usually located in the cortex; and those differentiated from adult neural stem cells (NSC) (18), which are found in the subventricular zone and adjacent to the corpus callosum, for example. The OPC population is heterogeneous and specific to particular brain regions, with remyelination efficiency depending on the origin of the OPCs (19). In the search for therapeutic strategies for remyelination, increasing attention has been paid to the potential role of OPCs. Transplantation offers several potential benefits: remyelination by the transplanted cells, stabilization of the demyelinated area, promotion of endogenous remyelination, and a potential neuroprotective mechanism involving growth factors secreted by the transplanted OPCs.

## USe of Undifferentiated Neural Stem Cells to Repair Multiple Sclerosis Lesions

NSCs can differentiate into neurons, astrocytes, and OLs. It has been suggested that they may be used to repair CNS lesions. Implantation of these cells has been shown to have beneficial effects on spinal cord lesions in rodent and primate models, supporting the use of the technique to treat MS (20). Nonetheless, the fact that MS is an autoimmune disease means that these cells' capacity to trigger immunogenicity is a significant consideration, which has led to a search for alternatives including the use of induced pluripotent stem cells (iPSC) (21). However, these cells frequently present genetic modifications, including aberrant DNA methylation and gene mutations, and it has been suggested that their use may lead to tissue aberrations or malignant transformation after transplantation (22, 23). The use of iPSCs bypasses ethical issues associated with embryonic or fetal stem cells, as they are generated from non-pluripotent cells. Another means of obtaining undifferentiated cells is the use of directly reprogrammed NSCs, which are generated from somatic cells and seem to be a safer alternative (24, 25). However, these cells appear inefficient in promoting remyelination in patients with MS, as only a small percentage of the NSCs grafted differentiate into OLs. One proposal to avoid this problem is the use of partially differentiated cells, such as bipotent glial cells capable of differentiating either into astrocyte or OL lineage cells, or OPCs, which are unipotent and can only differentiate into OLs. OPCs can be derived both from iPSCs (21, 26, 27) and from directly reprogrammed NSCs (28). Some researchers have even attempted to develop modified OPCs with an improved myelinogenic capacity.

Most evidence on the transplantation of OL lineage cells is from models of traumatic spinal cord lesions. These cells have been shown to be capable of promoting tissue repair and functional recovery (29, 30), with one study reporting that the implantation increased the number of OLs, resulting in improved motor function (31). While the mechanism behind this functional improvement is not known, it has been suggested that it may involve neurotrophic factors secreted by OPCs (32). OLs are highly susceptible to reduced survival due to the local cytotoxic conditions in and near immune and traumatic lesions. It has been reported that laboratory pre-differentiation of OL lineage cells and grafting of OPCs is more efficient for remyelination-mediated repair than the grafting of undifferentiated cells (33), particularly if the OPCs are enriched (34); this is a long process, however (35). The generation of OPCs from iPSCs is a recent development (36–38); other researchers have developed cell lines overexpressing such receptors as GPR17, promoting migration (39), or secreting platelet-derived growth factor-AA and fibroblast growth factor-2, favoring proliferation (40).

Human OPCs are known to promote remyelination (41), which has led to the use of various protocols in patients with spinal cord lesions (27, 31, 40–43), achieving an efficiency of differentiation into OLs of 40% of grafted cells. Administration of OPCs is also effective in rats with congenital hypomyelination (44) and stroke with white matter lesions (45). Research has shown that delayed cell transplantation is effective for older spinal cord lesions (46). The works of Prof Goldman's group highlight the potential of cell therapy in demyelinating pathologies, including those of genetic substrate (16, 47).

Most studies into cell therapies for MS do not use NSCs, as they aim to control the autoimmune mechanism rather than to promote remyelination. One proposed treatment is the transplantation of different cell types, including human embryonic cells, mesenchymal stem cells derived from human bone marrow (48), human placental stem cells (49), hematopoietic stem cells, human dental pulp stem cells (50), Wharton's jelly–derived stem cells (51), and undifferentiated adipose-derived stem cells (52); these cells have anti-inflammatory and immunomodulatory properties and can reduce degeneration in experimental autoimmune encephalomyelitis (53). However, the issue becomes more complex when we consider the efficiency of implantation *in vivo* (54) and whether the ability to promote myelin repair (55) owes more to the stimulation of an endogenous repair response or to the cells implanted and their neurotrophic function. These cells also promote remyelination and significantly reduce clinical signs of MS in an animal model of the disease. The most suitable route of administration is subject to debate. NSCs administered intravenously appear in the brain and spinal cord, proliferate, and migrate to MS lesions, probably due to chemotactic mechanisms (56). These findings were reported in various clinical trials, which are addressed in a recent review (57). OPCs, on the other hand, are able to migrate within the CNS but not from the cerebrospinal fluid or the bloodstream; therefore, the route of delivery is an important question if we have to use these cells as a treatment. One possibility is the nose-to-brain pathway, which has been used to administer drugs (58) and nanoparticles (59).

## Biomaterials in Cell Therapy

Biomaterials (BM) are natural or synthetic biocompatible materials used in the manufacture of devices that interact with biological systems. BMs are widely used in medicine. Applications include natural or synthetic polymers, used to treat wounds; drug delivery systems; vascular grafts; and tissue reconstruction (60). As well as being biocompatible, it is essential that BMs do not provoke adverse reactions after implantation, and that they continue functioning for the necessary period of time; this need has given rise to efforts to develop products with specific physical and chemical properties. For example, a BM may promote cell development and differentiation by creating a suitable local environment, improving the implanted cells' chances of survival (61). In the administration of OPCs to patients with MS, BMs may serve several purposes, enabling delivery of OPCs to the CNS and promoting migration to demyelinated areas, if they favor differentiation into OLs; nanofiber scaffolds resembling the natural structure of axons enable modeling of the interaction between axons and OLs, promoting neuron-glia interaction and myelination (62, 63). The use of biomaterials as particles is one of the most promising approaches. Particles constitute a transport system made up of natural or artificial polymers, enabling controlled, sustained delivery; specific targeting of lesions; and a high surface-area-to-volume ratio. This enables drugs to be administered at lower doses and frequency. Given these properties, particles may be the most suitable means of transporting cells (64).

**Figure 1:**
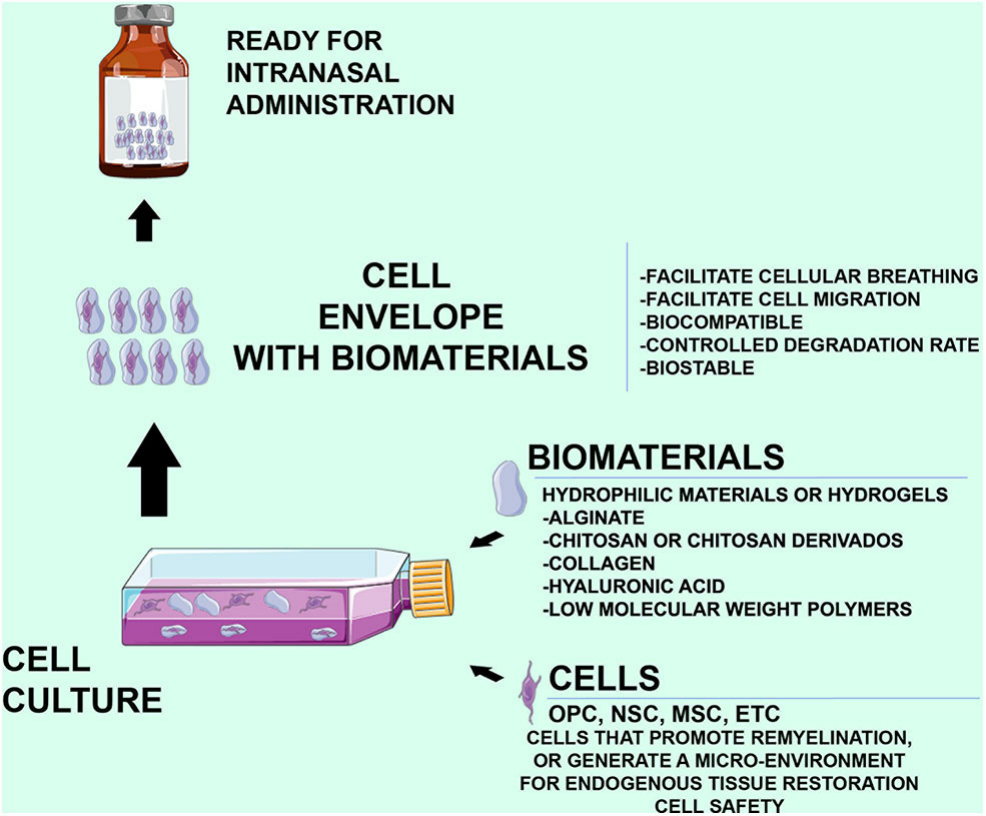
Characteristics of the biomaterials and cells in the intranasal administration, as well as the characteristics that must preserve the hydrogels that surround the cells. An Important fact is that the cells used should be safe without any possibility of generating tumor formations.

The specific BM chosen for cell therapy is an important factor, as the BM's surface properties are directly related to its biological behavior *in vitro* (e.g., adherence and the ability to permit cell proliferation and differentiation). Various natural materials, synthetic polymers, and ceramics have been proposed. Natural materials including purified collagen, hyaluronic acid, alginate, and chitosan have been used extensively in regenerative medicine and tissue engineering. Synthetic polymers are reproducible and can be modified to control their properties, such as degradation speed, mechanical properties, and shape. Calcium phosphate ceramics have been used in cell therapy for the skeletal system. Hydrogels constitute a particularly attractive class of materials (65). Hydrogels are networks of polymers, structured in a chemical or physical form, that expand in water and can be designed either with natural materials, such as alginate, or with synthetic polymers including polyethylene glycol. The specific advantages of these materials are the minimal adverse reactions in the host (i.e., biocompatibility), their high water content, the relatively mild reaction conditions, and the capacity for minimally invasive delivery as injectable vehicles. In the Figure 1, shows the characteristics that the cells, the biomaterials, as well as the attributes of the cells wrapped by biomaterials should have (Figure 1), acting as a fine wrap, to facilitate its release.

## Issues Related to the Administration of OPCs Through Particles to Promote Remyelination

OPCs clearly merit consideration as a therapeutic option targeting remyelination in MS, with the delivery of exogenous OPCs to demyelinated areas; however, there remain several outstanding issues (66). Many of these are addressed in an extensive review published recently by Unal et al. (67). Firstly, the most eligible patients are those with reduced remyelinating ability, specifically older patients or those with history of arterial hypertension (68), longer progression times, predominantly periventricular rather than subcortical lesions (periventricular lesions show lower levels of spontaneous remyelination), greater inflammatory activity (as measured with T2-weighted MRI studies), greater numbers of demyelinated areas (black holes on T1-weighted sequences) or larger areas of myelin loss (white matter PET scan) (69, 70), or pseudotumoral lesions that may hinder OPC migration (71). Additional relevant questions are: are exogenous OPCs sensitive to mechanisms regulating oligodendrogenesis, or do they behave in the same way as endogenous OPCs? Should chemical products be added to grafts as signals promoting remyelination? What effect does the coexistence of both endogenous and exogenous OPCs have on remyelination? Should the administration of OPCs be different in each patient, in accordance with the degree of demyelination/remyelination?

One important issue is the delivery of OPCs to the CNS and their migration to the lesion area. OPCs can only enter the CNS through direct delivery or through the nose-to-brain pathway, which is only possible if they are loaded into particles capable of crossing the nasal mucosa. Cells administered by this route travel directly to the CNS, with minimal loss to other regions (72). Particles should allow for migration to the lesion site and differentiation into OLs.

## Intranasal Rute

The basis of the mechanism of intranasal delivery is not understood, existent four main routes: [1] olfactory nerves, [2] trigeminal nerve pathway, [3] Lymphatic pathway, and [4] vascular pathways. Previously mentioned, the movement of molecules from the nasal cavity to the parenchyma of the brain occurs along both the nerve pathways (olfactory or trigeminal nerves) mainly, followed by vascular and lymphoid pathways. Dispersing throughout the brain, even reaching up to the cerebrospinal fluid (73). Two mechanisms are involved in this distribution: extracellular (used to transport large molecules and cells) and intracellular (used to transport by retrograde flow, small molecules, drugs, vectors, trophic factors, etc.). The extracellular pathway begins with the drug or cells crossing the nasal epithelium to the lamina propria, before being transported externally along the length of the neuronal axon by bulk flow processes. The axon leads into the CNS, where the drug or cells is distributed further via fluid movement. The intracellular mechanism starts with internalization of the molecule by an olfactory neuron, with the endocytic vesicle within the cell to the neuron's projection site (soma), it can be released by exocytosis or have its effect on the transport cell (74, 75).

Various materials have been used in the intranasal administration of drugs, vaccines or exosomes. In the Supplementary Material 1, a table is shown with the materials used in the intranasal administration of substances.

## Nasal Administration of Chitosan

Chitosan and its derivatives seem to be the most suitable natural polymers for administration to the CNS (76). Chitosan is a mucopolysaccharide closely resembling cellulose, produced during the deacetylation of chitin. It is derived from the shells of crustaceans and from fungal cell walls (77). Chitosan particles are biodegradable, biocompatible, and stable, with low toxicity, and are soluble in aqueous acid solutions (78, 79). While many routes of administration are available (80), nasal administration allows passage to the brain (81), and has been used for such drugs as anti-LINGO-1 (82), teriflunomide (83), and carbamazepine (84). Nasal administration of chitosan hydrogel has been used as a treatment for Alzheimer disease or biomedical applications in the CNS (85, 86). While there is less evidence on the transportation of cells than there is for drugs, chitosan is known to permit differentiation of both NSCs (87) and OPCs (88). Chitosan appears to be particularly appropriate for administration via the nose-to-brain pathway (89, 90). This route of administration is particularly attractive for the delivery of drugs to the CNS, as it bypasses the blood-brain barrier (91–93). The nasal cavity is connected to the brain via the olfactory and trigeminal pathways (94), enabling administration of drugs through the nasal mucosa (90). This is a painless, non-invasive means of administration and can be used to deliver therapeutic agents in patients with neurodegenerative diseases (95–99). Furthermore, drugs delivered through the nasal mucosa are not subject to hepatic first pass metabolism. Therefore, doses are usually 2–10 times lower if drugs are administered nasally rather than orally.

Up to one-third of patients with MS present olfactory alterations, although these are generally detected through physical examination rather than because of patients' complaints. Such alterations are attributed to changes in the connectivity of various CNS pathways, and are more frequently observed in patients with progressive forms of MS and with cognitive alterations (100); therefore, they should not affect the passage of particles to the brain.

If chitosan is to be used in treatments for MS, an autoimmune disease, we must consider whether the material provokes an immune response or triggers any kind of response favoring autoimmunity. However, chitosan has been used as a component of a vehicle for oral interferon beta in patients with MS (101), and for the administration of vaccines (102). It is also difficult to attribute immune responses exclusively to chitosan, as the products transported in the particles may themselves provoke such a reaction. Studies have shown that the treatment may provoke an innate (103) and adaptive immune response (104–106), although increased levels of anti-inflammatory cytokines have also been reported (107). Several syntethic polymers or natural biomaterials have been used for CNS applications, chitosan is nowadays one of the leading substrates, employed as it can be found in nature or as a modified derivative. In biomolecules delivery, it stands out for its mucoadhesive and BBB penetration enhancement properties that make it a great substrate for nose-to-brain approaches (76, 86, 97). For tissue engineering and regenerative medicine, chitosan and its derivatives have shown to promote axonal regeneration, anti-inflammation, and to successful deliver neurotrophic factors and cells with a consequently functional recovery (108). In this way, chitosan-based biomaterials have become increasingly popular to use, alone or in combination with other molecules (86).

**Figure 2:**
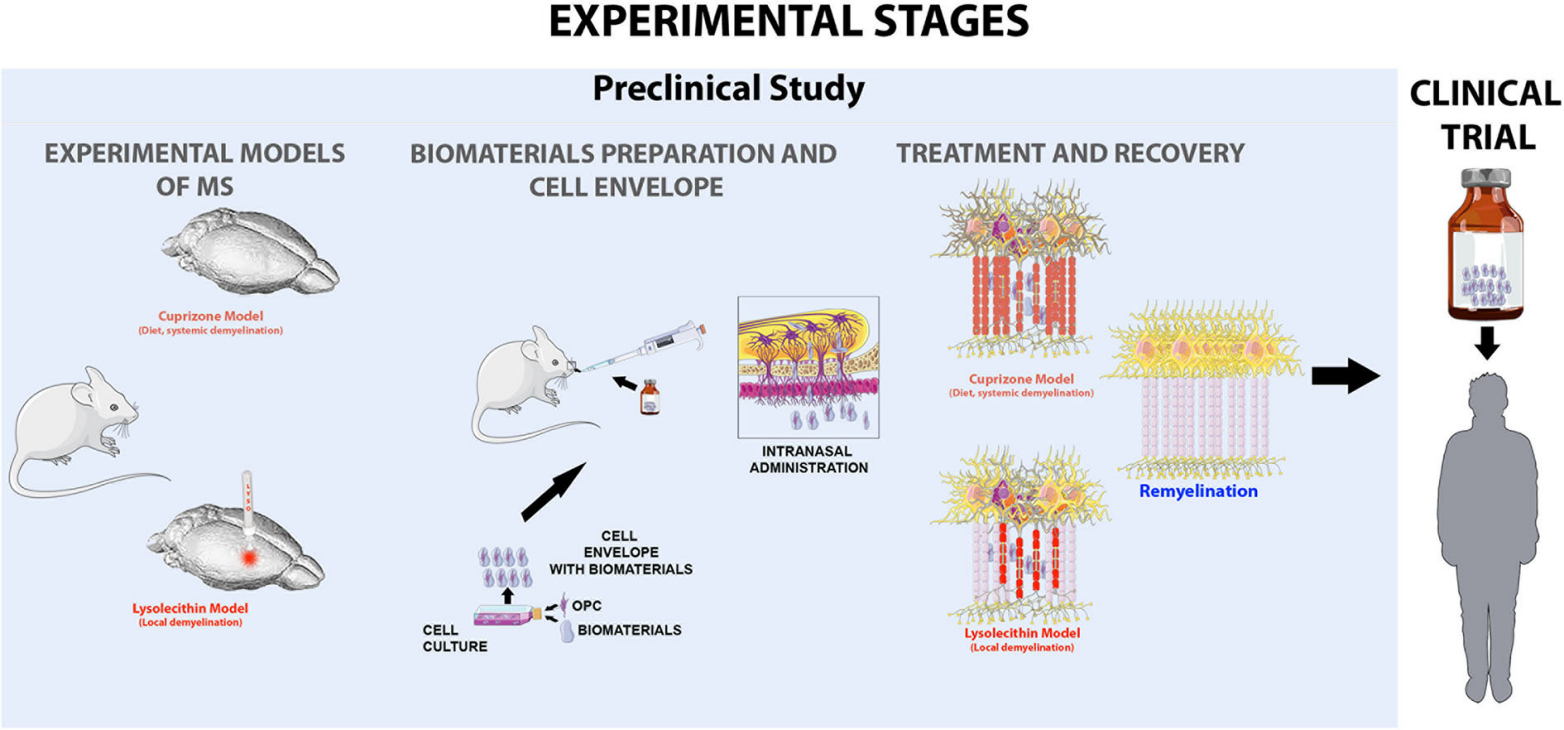
Experimental scheme proposed as a strategy to evaluate the viability of intranasal administration of cells in two models of multiple sclerosis, the cupriazone model (administered in the diet for 5 weeks) which is a systematic demyelination and the lysolecithin model, the which by means of the amplification by sterotactic injection in the corpus callosum of the animals induces foci of local demyelination. Where the main objective of our hypothesis is to achieve the activation of endogenous remyelination mechanisms in the central nervous system, favored by the application of myelinating cells wrapped in biocompatible biomaterials that preserve cell characteristics and allow to generate a favorable microenvironment.

In the Figure 2, shows in a schematic and simple way the stages in the development of the project, starting from the preclinical trial to the clinical trial (Figure 2).

## Conclusions

While the potential use of OPCs for remyelination is a promising therapeutic strategy, there is a need for basic research before clinical trials can be performed. It is also necessary to establish the best route of administration, although transporting cells in particles through the nose-to-brain pathway seems the most suitable. Researchers must also assess which BM is most appropriate; while chitosan and derivatives seem to be the most promising, we must assess the responses of patients with MS and whether migration to demyelinated areas is maintained. In conclusion, this line of research may yield an alternative to the remyelinating drugs currently being studied.

Studies should take into account various additional problems, including immune response to the treatment, given that MS is an autoimmune disease, and the use of associated immunomodulatory treatments. Treatment with repeated intracerebral injections of increasing doses of OPCs to different locations has been proposed as a treatment for patients with secondary-progressive MS. However, this method is not straightforward, given the need for administration protocols guaranteeing reproducibility and reducing the considerable rate of cell death associated with transplantation via injection; the survival rate can reach 1% due to such factors as exposure of cells to an inflammatory microenvironment, limited diffusion of oxygen and nutrients, immune destruction, dispersion through a deteriorated local vascular system, and activation of apoptosis and autophagy.

## Author Contributions

JM-G and UG-P: lead researchers. All authors: manuscript drafting, research project group, and critical review of the manuscript.

## Conflict of Interest

JM-G receives honoraria from the journal *Neurologia* as editor-in-chief and has received personal consultancy fees from Lilly. The remaining authors declare that the research was conducted in the absence of any commercial or financial relationships that could be construed as a potential conflict of interest.

## Supplementary Material

Table 1 Materials used in intranasal administration, show their topical form (spay, particles, gel) and the therapeutic strategy.
