# Supplementary material for: Particles Containing Cells as a Strategy to Promote Remyelination in Patients With Multiple Sclerosis
Source: Front Neurol. 2020 Jul 7;11:638. doi: 10.3389/fneur.2020.00638 (PMC7358567; doi:10.3389/fneur.2020.00638)
Supplement: Supplementary file 1 [file Data_Sheet_1.PDF]

Chitosan	Microspheres	Nasal Vaccine Carriers	Islam MA, *Int J Nanomedicine*. 2012;7: 6077–6093. doi:10.2147/IJN.S38330
	Gels and Nanoparticles	Nasal Delivery Systems for Therapeutic Drugs	Casettari L. *J Control Release*. 2014;190:189–200. doi:10.1016/j.jconrel.2014.05.003
	Nanoparticles	Nasal Exosomas Delivery	Dong X. *Theranostics*. 2018;8(6):1481–1493. Published 2018 Feb 5. doi:10.7150/thno.21254
Hyaluronic Acid	Nanoparticles	Nasal Vaccine Carriers	Fan Y. *J Control Release*. 2015;208:121–129. doi:10.1016/j.jconrel.2015.04.010
Gellan	Nanoparticles	Nasal Vaccine Carriers and Drugs Delivery Systems	Bacon A. *Infect Immun*. 2000;68(10):5764–5770. doi:10.1128/iai.68.10.5764-5770.2000
Peptide Arginine-Glycine-Aspartic Acid (RGD)	Nanoparticles	Drugs Delivery Systems	Jansson B. *Eur J Pharm Biopharm*. 2005;59(3):557–564. doi:10.1016/j.ejpb.2004.10.001
Poly Lactic-co-Glycolic Acid (PLGA)	Nanoparticles	Anti-inflammatory Drugs	Cayero-Otero MD. Curr Pharm Des. 2018;24(14):1589–1616. doi:10.2174/1381612824666180403113015
	Microspheres	Allergy Immunotherapy	Reisacher WR. *Curr Opin Otolaryngol Head Neck Surg*. 2011;19(3):188–192. doi:10.1097/MOO.0b013e328345013a
Polylactic acid (PLA)	Nanoparticles	Drug Delivery Systems	Tyler B. *Adv Drug Deliv Rev*. 2016;107:163–175. doi:10.1016/j.addr.2016.06.018
	Nanoparticles	Neuroprotective Molecules or Proteins	Giordano C. *Int J Artif Organs*. 2011;34(12):1115–1127. doi:10.5301/IJAO.2011.8915
Starch	Nanoparticles	Nasal Vaccine Carriers	Moreno-Mendieta SA. *Int J Pharm*. 2014;474(1-2):241–248. doi:10.1016/j.ijpharm.2014.07.041
Alginate	Nanoparticles	Nasal Vaccine Carriers and Drug delivery systems	Sarei F. Indian J Pharm Sci. 2013;75(4):442–449. doi:10.4103/0250-474X.119829
